# Exploration of the Transglycosylation Activity of Barley Limit Dextrinase for Production of Novel Glycoconjugates

**DOI:** 10.3390/molecules28104111

**Published:** 2023-05-16

**Authors:** Malene Bech Vester-Christensen, Jesper Holck, Martin Rejzek, Léa Perrin, Morten Tovborg, Birte Svensson, Robert A. Field, Marie Sofie Møller

**Affiliations:** 1 Enzyme and Protein Chemistry, Department of Biotechnology and Biomedicine, Technical University of Denmark, DK-2800 Kongens Lyngby, Denmark; mbxc@novonordisk.com (M.B.V.-C.); bis@bio.dtu.dk (B.S.); 2 Enzyme Technology, Department of Biotechnology and Biomedicine, Technical University of Denmark, DK-2800 Kongens Lyngby, Denmark; jesho@dtu.dk; 3 Department of Biological Chemistry, John Innes Centre, Norwich Research Park, Norwich NR4 7TJ, UK; martin.rejzek@jic.ac.uk (M.R.); robert.field@manchester.ac.uk (R.A.F.); 4 Applied Molecular Enzyme Chemistry, Department of Biotechnology and Biomedicine, Technical University of Denmark, DK-2800 Kongens Lyngby, Denmark; lea.perrin42@yahoo.com; 5 Novozymes A/S, DK-2800 Kongens Lyngby, Denmark; motj@novozymes.com

**Keywords:** glycoside hydrolase family 13, α-glucan debranching enzyme, CAZyme profiling, pullulan, cyclodextrins, glycosyl fluorides

## Abstract

A few α-glucan debranching enzymes (DBEs) of the large glycoside hydrolase family 13 (GH13), also known as the α-amylase family, have been shown to catalyze transglycosylation as well as hydrolysis. However, little is known about their acceptor and donor preferences. Here, a DBE from barley, limit dextrinase (*Hv*LD), is used as a case study. Its transglycosylation activity is studied using two approaches; (i) natural substrates as donors and different *p*-nitrophenyl (pNP) sugars as well as different small glycosides as acceptors, and (ii) α-maltosyl and α-maltotriosyl fluorides as donors with linear maltooligosaccharides, cyclodextrins, and GH inhibitors as acceptors. *Hv*LD showed a clear preference for pNP maltoside both as acceptor/donor and acceptor with the natural substrate pullulan or a pullulan fragment as donor. Maltose was the best acceptor with α-maltosyl fluoride as donor. The findings highlight the importance of the subsite +2 of *Hv*LD for activity and selectivity when maltooligosaccharides function as acceptors. However, remarkably, *Hv*LD is not very selective when it comes to aglycone moiety; different aromatic ring-containing molecules besides pNP could function as acceptors. The transglycosylation activity of *Hv*LD can provide glycoconjugate compounds with novel glycosylation patterns from natural donors such as pullulan, although the reaction would benefit from optimization.

## 1. Introduction

Glycoside hydrolase family 13 (GH13), also known as the α-amylase family, is the largest GH family in the Carbohydrate-Active enzymes (CAZy) database (www.cazy.org (accessed on 9 May 2023); [1]). This family includes over 153,800 protein sequences assigned to 31 different activities, including both hydrolase and transglycosidase activities, targeting α-glucosidic linkages. GH13 is further divided into 46 subfamilies to reflect the large diversity within this family [1,2]. Many GH13 members possess both hydrolytic and transglycosylation (TG) activity, though usually with a clear preference for one of them, which is also observed among the GH13 α-glucan debranching enzymes (DBEs) belonging to subfamilies 11–14. These enzymes’ primary activity is to hydrolyze α-1,6-glucosidic linkages, but some also have TG activity, in which small oligosaccharides are transferred from one molecule to another, leading to the formation of an α-1,6-glucosidic linkage. This reaction is typically driven towards TG rather than hydrolysis at high substrate concentrations. TG activity has been studied for over five decades, as it can result in products that are challenging or impossible to obtain by classical organic synthesis [3]. Additionally, the addition of glucose units to non-carbohydrate compounds such as secondary metabolites can modify their properties, including increased water solubility due to the high polarity of the sugar moiety, thus improving their bioavailability and potency [4,5,6]. GH13 enzymes can primarily form α-1,4- or α-1,6-glucosidic linkages by TG. The ability of the DBEs to form α-1,6-glucosidic linkages is of particular interest since α-1,6-glucosidic linkages possess unique properties when compared to α-1,4-glucosidic linkages, including greater flexibility due to the methylene group of C6 and different solubility resulting from the ability to form a greater number of hydrogen bonds with water [7]. Additionally, compounds with a particular pattern of α-1,6-glucosidic linkages are resistant to degradation or are only partially degraded in the upper human gastrointestinal tract, making them available for utilization by the microbiota and potentially serving as prebiotics [8,9,10,11,12].

**Figure 1:**
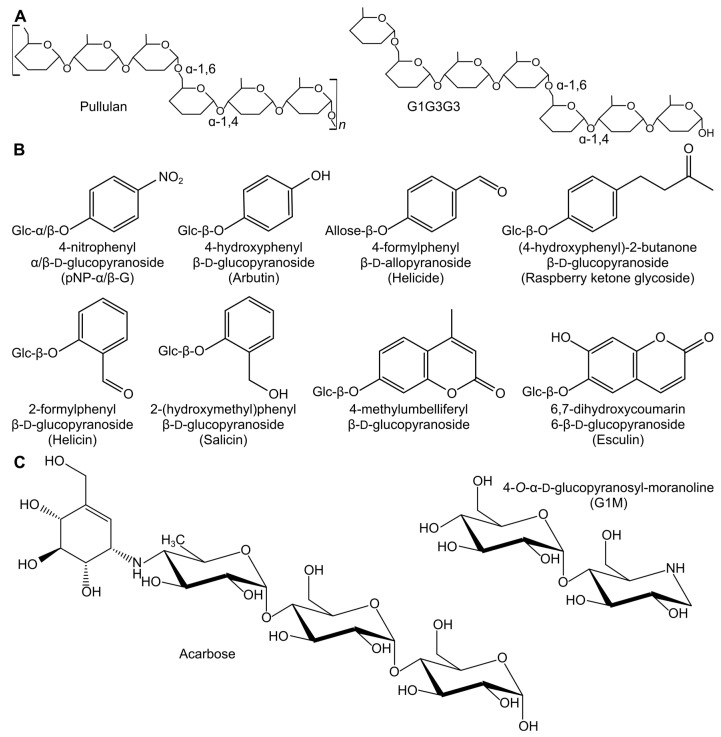
Donors and acceptors for TG reactions. (**A**) Schematic drawings of the natural donors. (**B**) Structure of the aglycone part of the glycoside compounds tested as acceptor/donors. The position of the glucose (Glc)/allose units and the linkage type is indicated. (**C**) Structures of the GH inhibitors used as acceptors with G2F.

Among the DBEs, the industrially relevant type I pullulanases (PULIs) belonging to GH13 subfamilies 12–14 have attracted particular attention. *Bacillus acidopullulyticus* and *Klebsiella pneumoniae* PULIs have been reported to produce mono- and di-maltosyl α-1,6-substituted cyclodextrins (CDs) using α-maltosyl fluoride (G2F) as donor and α- and β-CDs as acceptors in TG reactions [13,14,15,16]. Here, the strongly activated G2F mimics the reaction intermediate of retaining GHs. Glycosyl fluorides have generally been used as glycosyl donors with many different GHs [17]. Additionally, a PULI from the thermophilic bacterium *Thermotoga neapolitana* has been shown to have α-1,6 transfer activity. It can form α-maltotriosyl-1,6-esculin in a reaction with pullulan, a polysaccharide consisting of maltotriose (G3) connected by α-1,6-glucosidic linkages (Figure 1A), and the coumarin glucoside esculin (Figure 1B) [18]. Furthermore, a *Klebsiella* PULI can use the natural sweetener stevioside as an acceptor with pullulan as donor [19]. Hence, small glycoside compounds can function as acceptors in TG reactions of PULIs.

McDougall et al. showed that a GH13 subfamily 13 PULI from barley, limit dextrinase (*Hv*LD), exhibits TG activity. When activity was measured using the Limit Dextrizyme assay (azurine-crosslinked-pullulan), they observed that linear maltooligosaccharides ranging from maltose to maltoheptaose (G2–G7) increased the activity of *Hv*LD until a concentration of 5–12.5 mM, depending on the length of the maltooligosaccharide. Above this concentration, *Hv*LD activity was inhibited. This activation was also observed when Red Pullulan (Procion Red-crosslinked-pullulan) was used as a substrate and G3 as an activator [20]. These results confirmed the previous findings by MacGregor et al. that maltodextrins derived from malt extract activated *Hv*LD when activity was assayed using dyed pullulan substrates, whereas no activation was observed when β-limit dextrin, a natural substrate, was used for assaying activity [21]. Furthermore, McDougall et al. used an assay with pullulan as substrate and a fluorescent G3 derivative (maltotriose-8-amino-1,3,6-pyrene trisulphonic acid; G3-APTS) as activator, resulting in *Hv*LD catalyzing the formation of APTS-containing products of a greater molecular weight. *Hv*LD also degraded these products with the formation of fluorescent oligosaccharide derivatives. Similarly, rice limit dextrinase was activated by linear maltodextrins and also catalyzed TG, while bacterial PULIs from *B. acidopullulyticus* and *Klebsiella planticola* did not catalyze TG with G3-APTS [20], emphasizing that the TG activity is enzyme dependent. Furthermore, 4,6-*O*-benzylidene-4-methylumbelliferyl β-6^3^-α-maltotriosyl-maltotrioside (BzMUG_3_G_3_) could take part in a TG reaction by *Hv*LD when G2 was present, while no TG was seen for a similar substrate with the chromogenic group 2-chloro-4-nitrophenyl (BzCNPG_3_G_3_) replacing the fluorescent moiety [22]. In addition, a *Klebsiella* PULI was used to produce fluorogenic branched dextrins from pyridylaminated maltooctaose and maltotetraose [23].

To probe the TG activity of *Hv*LD and the involved subsites of the enzyme, we used two approaches; (i) natural substrates as donors (Figure 1A) and different pNP-sugars as well as different small-molecule glucosides as acceptors (Figure 1B), and (ii) G2F and α-maltotriosyl fluoride (G3F) as donors and a range of linear oligosaccharides (G2–G6), CDs (α-CD, β-CD, and G2-β-CD), and glycoside hydrolase inhibitors as acceptors. TG product formation followed, using thin-layer chromatography (TLC) combined with product identification by various mass spectrometry methods.

## 2. Results and Discussion

### 2.1. Transglycosylation Reactions with Natural Substrates and pNP-Sugars

pNP-sugars are known to function as donors in the TG reaction of different GHs due to pNP being a potent leaving group, and the TG activities of GH13 enzymes were used early on for producing synthetic substrates such as *p*-nitrophenyl (pNP) maltooligosaccharides for assaying the activities of GH13 enzymes [24]. Hence, the ability of *Hv*LD to perform TG with pNP-sugars (pNP-α-G, pNP-β-G, pNP-G2, pNP-G3, and pNP-G4) was investigated. Two different experimental series were conducted: (i) reactions including only the pNP-sugars and *Hv*LD, and (ii) reactions including both pNP-sugars and either pullulan or a pullulan fragment (6^3^-α-(6^3^-α-D-glucosyl-maltotriosyl)-maltotriose; G1G3G3; Figure 1A) with *Hv*LD. The identification and quantification of TG products from reactions including pNP-sugars were performed via liquid chromatography electrospray ionization mass spectrometry (LC-ESI-MS). TG products were observed and identified as deprotonated and formate adducts ([M-H]^−^ and [M+formate]^−^). Compounds with 2–5 glucose units were observed as single charge, whereas longer structures were observed as double charge. No pNP-sugars besides starting materials were observed in negative controls without enzyme. TG products were identified by mass-to-charge ratio (*m/z*) and MS^2^ fragmentation, if possible. The fragmentation pattern was a combination of C-, B-, and X-type fragments, including the ^0,4^A-type fragment (C+42) (nomenclature follows [25]) that has previously been reported to be typical for α-1,6-linked glucose [26] (see Figure S1 for example).

**Figure 2:**
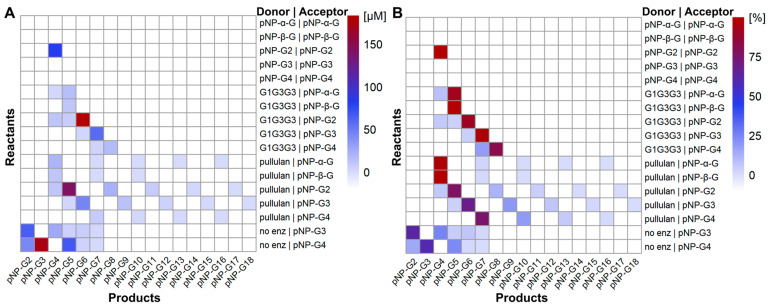
TG products from *Hv*LD identified by LC-ESI-MS and quantified by UV absorbance. (**A**) Total amounts of product formed after 2 h reaction expressed as pNP-G2 equivalents. (**B**) Data normalized to percentage of observed products in the sample. The major starting compounds are not included in the heat maps. Based on the analysis method, the position of the α-1,6 linkages cannot be determined; hence, the products differ in branch pattern while having the same mass. No UV detectable products were identified for control reactions including only G1G3G3 or pullulan. For reaction conditions and LC-ESI-MS analysis description, see Section 3.2.

For reactions containing only pNP-sugars, only pNP-G2 could function as both donor and acceptor, resulting in the formation of pNP-G4 (Figure 2). The TG product (pNP-G2-G2) was also a substrate for the hydrolysis reaction by *Hv*LD, as G2 was formed over time along with a decay in the TG product (Figure S2). No TG products were observed in reactions containing pNP-α-G or pNP-β-G. Analysis of the reactions containing pNP-G3 or pNP-G4 revealed several pNP-sugars in the range from pNP-G2 to pNP-G7. However, these structures were not a result of TG reactions, but impurities already present in the substrates (Figure 2), and they were hence subtracted from the results.

Then, reactions including pullulan or G1G3G3 (Figure 1A) and the different pNP-sugars were performed. Pullulan has been shown to be able to serve as donor for TG reactions with PULIs [18,19]. The pullulan fragment G1G3G3 could serve as donor in *Hv*LD TG reactions with all five pNP-sugars tested as acceptors (Figure 2). The major products had masses equal to the transfer of four glucose units to the pNP acceptors; however, smaller products were observed in all cases, except for pNP-β-G. The products deviating from the addition of four glucose units could stem either from impurities of the donor substrate or from TG reactions, where the pNP-sugars were functioning as both donors and acceptors. Again, pNP-G2 was the best acceptor, followed by pNP-G3.

**Figure 3:**
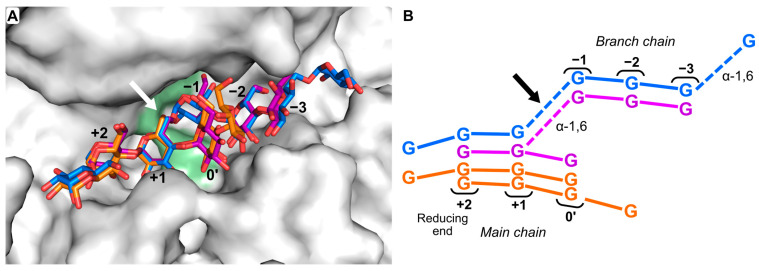
Subsites of *Hv*LD. (**A**) Close-up of the active site of *Hv*LD (Protein Databank, PDB, entry 4J3T) soaked with G4 (orange sticks; two overlapping G4 molecules). The structure is superimposed with ligands from two other *Hv*LD structures; G1G3G3 (blue sticks; PDB entry 4J3X) and a branched limit dextrin (purple sticks; PDB entry 4J3W). The position of the catalytic residues is shown in green and the position of the α-1,6 cleavage site is indicated by an arrow. (**B**) Schematic view of the ligands included in (**A**) with well-defined main- and branch-chain subsites designated. α-1,4-glycosidic linkages are shown as solid lines, while α-1,6-glycosidic linkages are shown as dotted lines. The α-1,6 cleavage site is indicated by an arrow.

When pullulan was used as donor, TG products were observed with all pNP compounds tested (Figure 2). The major products in each case were equal to the transfer of one G3 unit to the pNP acceptor (Figure 2B). This was expected, as G3 is the major product from *H*vLD hydrolysis of pullulan. In addition, a product with a mass equal to pNP-G4 with pNP-G2 as acceptor was observed. Hence, pNP-G2 also functions as donor. Interestingly, in all cases, longer products following a plus-three-glucose-units pattern were observed, though at relatively lower concentrations. They are either produced in a one-step reaction by the transfer of longer fragments from the pullulan or in several reactions by the stepwise transfer of G3 units to the TG products. Both scenarios are possible for pullulan. No longer TG products were observed with G1G3G3, hence, here, stepwise extension is not possible. The TG products from G1G3G3 could be worse acceptors for *Hv*LD, as they likely do not bind as well in the acceptor subsites +1 and +2 due to the α-1,6 linkage next to the terminal glucose unit (GM3; Figure 3). The observation of TG products for reactions with pNP-G3 or pNP-G4 and pullulan or G1G3G3 as donors implies that the absence of products in reactions with only pNP-G3 or pNP-G4 was due to poor donor capability, rather than lack of acceptor capability (Figure 2). Based on our analytical methods, we cannot determine the position of the formed branches, i.e., in the case of pNP-G2 to pNP-G4. For the intermediate-sized TG products, i.e., degree of polymerization of 5–7, both UV and extracted ion chromatograms showed dual peaks, indicating more than one structural isomer (Figure S3). It is possible that the branch is added to another glucose unit than the terminal one since *Hv*LD has one well-defined additional main chain-binding subsite (subsite 0′) (Figure 3). This subsite can accommodate at least one more main chain glucose unit to the non-reducing site of the branch. Furthermore, a complex structure between *Hv*LD and G4 shows that an additional glucose unit can be accommodated next to the one in subsite 0′ (Figure 3B).

Based on a control experiment, no TG products were observed, when G3 was present instead of pullulan together with pNP-G2. Hence, the products in the reactions with natural substrates as donors most likely originate from a transfer reaction rather than a condensation reaction, which some PULIs are known to be able to perform at high substrate concentrations and over very long reaction durations (several days) [27,28].

Notably, pNP-glucosides usually function as donors in TG reactions with GHs due to pNP being a good leaving group [29], and pNP-glucosides are usually good model substrates for many GHs, but not for DBEs. There is only one case where a pNP-maltooligosaccharide has been reported as a substrate for a PULI; namely, pNP α-maltopentaoside as a substrate for a PULI from *Streptococcus pneumonia* (Michaelis–Menten constant, *K*_M_ = 360 µM; turnover number, *k*_cat_ = 270 min^−1^) [30]. However, with *Hv*LD, the pNP-sugars function as acceptors in reactions including natural substrates. Pullulan and G1G3G3 bind perfectly in both the plus and minus subsites (Figure 3), while the positioning of the pNP motif in the subsite +1 is less optimal because the distance between the pNP group and its glucosides is shorter than the α-1,6 linkage of the natural substrates. Altogether, this makes the natural substrates donors, despite pNP being a very efficient leaving group in the model substrates.

### 2.2. Alternative Compounds as Acceptors

To investigate the influence of the aglycone structure of an acceptor glycoside on the ability of *Hv*LD to utilize it in a TG reaction, six additional β-glycoside compounds and one alloside compound (Figure 1) were tested with G1G3G3 as the donor substrate. Surprisingly, all compounds were found to function as acceptors, resulting in the formation of compounds with a UV spectrum comparable to the starting material, but with *m/z* values corresponding to the respective glycoside with four glucose units added (Figure S4). The aglycone part of the compounds must be positioned in the subsite +2, which is considered the high-affinity site, while the glucose or allose unit must bind in subsite +1. Notably, esculin, which was among the tested compounds, has been previously shown to act as an acceptor in a TG reaction with pullulan as donor catalyzed by a PULI from *T. neapolitana* [18].

*Hv*LD has the ability to use pullulan and G1G3G3 as donors for reactions with various aglycone glucosides including pNP-sugars, resulting in the formation of novel glycoconjugates. The utilization of natural substrates as donors is compelling. However, the optimization of the reaction conditions could increase the yields, as TG is kinetically controlled [17]. Yields could be increased by varying the ratios of donor and acceptor, reaction times, and *Hv*LD concentrations. Another approach to increase yields could be through the mutation of selected residues in the active site, either the nucleophile to obtain a glycosynthase [31], or an evolutionary approach involving the mutation of conserved residues but excluding catalytic residues [32,33].

### 2.3. Probing the Acceptor and Donor Length Preferences Using Fluoride Maltooligosaccharides

The TG activity and acceptor length preferences of *Hv*LD were further investigated using G2F and G3F as donors, and linear maltooligosaccharides (G2; G3; maltotetraose, G4; and maltohexaose, G6) and cyclic oligosaccharides (α-CD, β-CD, and G2-β-CD) as acceptors.

**Figure 4:**
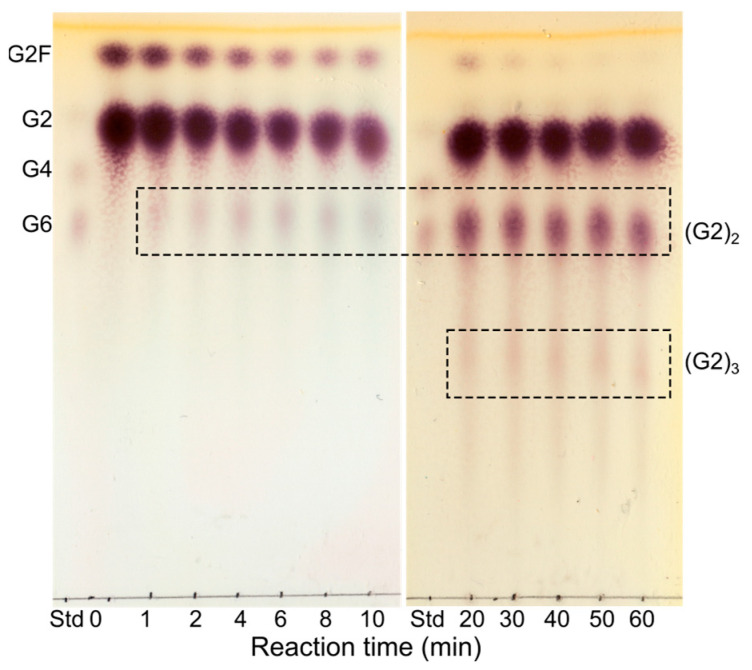
TLC analysis of the reaction with G2F as donor and G2 as acceptor. TG products are framed and annotated based on MALDI-TOF MS analysis (see MS spectrum in Figure S5). Maltooligosaccharides (G2, G4, and G6) are included as standards (Std).

When G2 was used as an acceptor, the conversion rate of G2F in transglycosylating the acceptor was high, as indicated by a fast decrease in the amount of G2F. The subsequent hydrolysis of the formed product seemed to be relatively slower compared to that of TG, as indicated by the accumulation of TG product (Figure 4). The major product formed was (G2)_2_. However, the products formed from the initial TG also acted as acceptors resulting in a polymerization of G2 units with up to eight G2 α-1,6-linked repeats, which were identified by matrix-assisted laser desorption/ionization time-of-flight (MALDI-TOF) MS (Figure S5). Based on previous NMR results [34], the G2 unit of G2F is expected to be transferred to the non-reducing end of G2. Furthermore, the strong interaction at subsite +2 mediated by hydrophobic stacking and hydrogen bonds [35] presumably contributes to the majority of the α-1,6 linkages being formed at the non-reducing end glucose unit. This is supported by the fact that a pullulanase from *Aerobactor aerogenes* has been shown to add G2 to both the reducing and non-reducing ends of a G2 acceptor in a condensation reaction, with the majority of the products (86%) having the α-1,6 linkage at the non-reducing glucose unit [27].

The conversion rate of G2F to longer linear oligosaccharides (G3–G6) was slower than to G2, and the subsequent hydrolysis of the formed product was relatively faster, resulting in only minor accumulation of TG product and an increase in G2 concentration.

**Figure 5:**
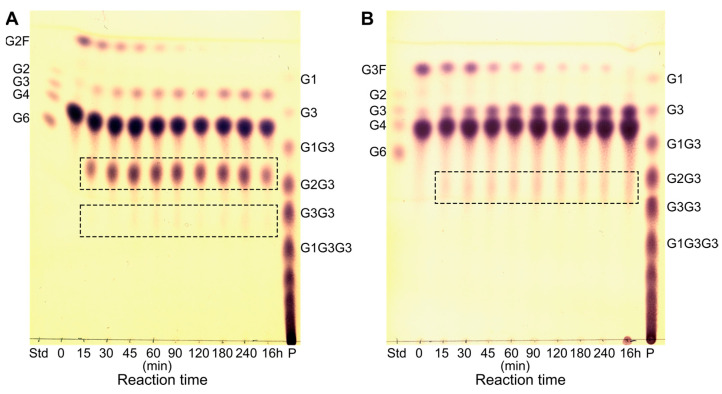
Comparison of the TG reaction using either maltosyl-fluoride (G2F) (**A**) or maltotriosyl-fluoride (G3F) (**B**) as donor and G4 as acceptor. Donor/acceptor ratio 1:2 (20 mM: 40 mM). TG products are framed. Maltooligosaccharides (G2, G3, G4, and G6) are included as standards (Std) together with a pullulan standard (P).

With G4 as an acceptor, the conversion rate of G3F was slower than that of G2F, based on the observed slower decrease in donor amount (Figure 5). Furthermore, very low accumulation of TG product was observed with G3F as a donor (Figure 5B). It is likely that the formed TG product is a better glycosyl donor than G3F, leading to a fast hydrolysis of the TG product. A higher hydrolysis rate of the TG product from the reaction with G3F as donor and G4 as acceptor could also explain the apparent difference between G2F and G3F in the ability to function as an activated substrate donor.

The use of G4 as an acceptor with G2F or G3F as donors can, in theory, lead to four different products, with one G2/G3 unit substituted to any one of the four glucose residues. However, not all four glucose units of G4 are equally likely to be in a position to accept the side chain. The position of the side chain is most likely on one of the middle glucose units; in the crystal structures of *Hv*LD in complex with G4 (PDB entries 4J3S and 4J3T), the G4 clearly binds at subsites +2, +1, and 0′. Then, the fourth residue can be accommodated at either side of the main chain subsites, extending beyond the +2 or the 0′ subsite (Figure 3) [35]. Furthermore, the results from the TG with different lengths of donor and acceptor clearly show that longer sugar chains, both side and main chain, are preferred for hydrolysis. These results are in agreement with previous analyses of the hydrolytic specificity of *Hv*LD towards small-branched oligosaccharides: substrates with at least one glucose unit at both sides of the glucose unit with the branch have lower *K*_m_ values and faster turnover numbers than those with the α-1,6 linkage on the non-reducing end [34,36].

**Figure 6:**
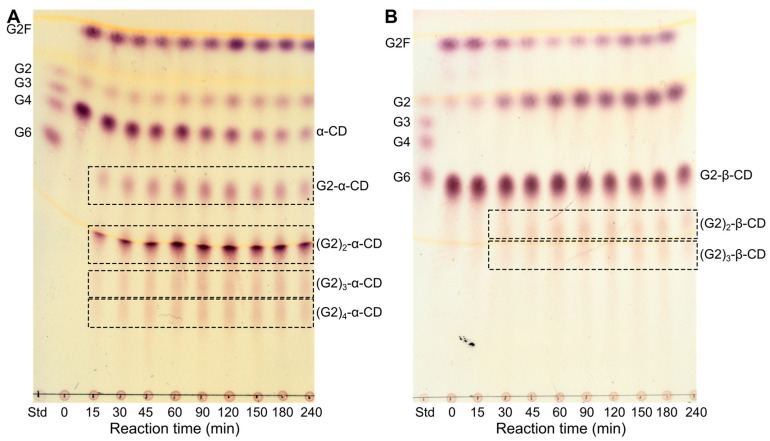
Comparison of the TG reaction using G2F as donor and either α-CD (**A**) or G2-β-CD (**B**) as acceptors. Donor/acceptor ratio of 4:1 (40 mM:10 mM). G2F was added to the reaction at different time points to maintain a constant level of G2F. TG products are framed. See Figure S6 for MS identification of TG products from the G2F–α-CD reaction. Maltooligosaccharides (G2, G3, G4, and G6) are included as standards (Std).

Using α- and β-CD, which are known inhibitors of *Hv*LD binding at subsite +2 [37,38], and 6-*O*-α-maltosyl-β-CD (G2-β-CD) as acceptor and G2F as donor, multiple substitutions, presumably of the ring glucosyl residues, were observed both via TLC and MS. These experiments were carried out with a large excess of the donor (donor/acceptor ratio of 4:1; 40 mM:10 mM) in the reaction mixture, and additional G2F was added to the reaction at different time points to maintain a constant level of G2F. The constant level of G2, originating from G2F, and the clear decrease in α-CD indicate that the product formed in the conversion of G2F to α-CD was not hydrolyzed (Figure 6A), and under these reaction conditions, six substitution products ((G2)_1–6_-α-CD) were detected by MS (Figure S6). Though the MS data cannot determine if the G2s are positioned on the CD ring and/or if further polymerization occurs of the branches of the TG products, the synthesis of multiple substituted products (dimaltosyl-β-CD and trimaltosyl-β-CD) by *K. pneumonia* PULI-catalyzed TG has been previously reported [14,16]. Despite a four-fold excess of donor, no products from G2 polymerization were observed, indicating that the CDs are much better acceptors than G2 for the TG reaction. This conforms with the high affinity of *Hv*LD towards the CDs (*K*_d_ of α- and β-CD is 27.2 and 0.7 μM, respectively [37]). The TG reaction with maltosyl-β-CD (G2-β-CD) as acceptor seems to be slightly faster than with α-CD as acceptor. However, the subsequent hydrolysis of the product seems to be even faster than the TG, resulting in only a minor accumulation of the TG product (Figure 6B). The same result was observed when using β-CD as acceptor (Figure S7).

The isomaltooligosaccharide structures obtained from the *Hv*LD-catalyzed TG may be prebiotic candidates [12]. Furthermore, the products from the reactions with CDs as acceptors could function as drug carriers or as food ingredients [39,40,41]. However, the need for fluoride oligosaccharides as donors limits the feasibility of the reaction for industrial-scale production.

### 2.4. Production of Potential GH Inhibitors from Known GH Inhibitors

In addition to the linear and cyclic oligosaccharides, the known GH inhibitors, 4-*O*-α-D-glucopyranosyl-moranoline (G1M) and acarbose (Figure 1C) [42,43], were also used as acceptors with G2F as donor in order to produce new inhibitors. The reactions primarily resulted in the polymerization of G2. However, MS data revealed the formation of TG products with the unique molecular masses of single and double G2 substitutions of both acceptors (Figures S8 and S9). The inhibition ability of G1M and other iminosugars is believed to be caused by mimicking both the conformation and the charge of the oxocarbonium ion enzyme intermediate [44], involving the binding of G1M at subsites –1 and –2 of the active site. This exact binding mode was seen for a PULI from *S. pneumoniae* (PDB entry 2YA2) [30]. However, the TG product formation from the transfer of G2F to G1M suggests that G1M can also bind at *Hv*LD subsites +1 and +2. Based on the previous results with the transfer of G2F to G2, it is most likely that the transfer of G2F to G1M occurs on the glucopyranose ring, which is assumed to be positioned at subsite +1.

Similarly to G1M, acarbose has been shown to bind in the branch subsites −1, −2, and −3 in complexes with two different GH13 DBEs; glycogen debranching enzymes from *Sulfolobus solfatarius* [45] and *Streptomyces* [46]. Like G1M, acarbose must also be able to bind to the main chain subsites of *Hv*LD to act as an acceptor. Isolation and structural analysis of the products would be necessary to determine the position of the newly formed α-1,6 branch point.

The TG products from the transfer of G2 to G1M and acarbose by *Hv*LD could potentially be inhibitors with altered inhibitory activity towards GHs compared to G1M and acarbose. However, similar to the novel glycoconjugates obtained with natural substrates as donors, optimization of the TG reactions with G1M and acarbose as acceptors is needed to increase the yield of the products.

## 3. Materials and Methods

### 3.1. Materials

Pullulan and G1G3G3 were purchased from Megazyme Co. Ltd. (Wicklow, Ireland). pNP-α-G, pNP-β-G, pNP-G2, and pullulan standard 1,300 were from Sigma-Aldrich (Darmstadt, Germany), while pNP-G3 and pNP-G4 were from Boehringer Mannheim (Mannheim, Germany). Arbutin, salicin, raspberry ketone glucoside, esculin, 4-methylumbelliferyl β-d-glucoside, 4-formylphenyl β-d-allopyranoside, and 2-formylphenyl β-d-glucopyranoside were from Biosynth Ltd. (Compton, UK). G2F and G3F were prepared following a procedure previously published [47]. *Hv*LD was recombinantly produced and purified as previously described [37].

### 3.2. Analysis of Transglycosylation Activity with Glucoside Compounds and Natural Substrates

To address *Hv*LD reactivity with pNP compounds (pNP-α-G, pNP-β-G, pNP-G2, pNP-G3, and pNP-G4), an analysis was conducted: 50 µL reaction mixture including 14 mM pNP compound (except for pNP-α-G, where the concentration was 8 mM due to limited solubility) and 50 nM *Hv*LD in 20 mM sodium acetate pH 5.5, 5 mM CaCl_2_, was incubated at 37 °C for 2 h followed by 10 min at 95 °C to terminate the reaction. Likewise, reactions including pullulan or G1G3G3 as donors and the pNP compounds as acceptors were performed as described above, but 10 mg/mL pullulan or G1G3G3 was included in the reaction mixture. In addition, a two-step control to test for condensation activity was conducted by initially treating 25 µL 20 mg/mL pullulan with 50 nM *Hv*LD in 20 mM sodium acetate pH 5.5, 5 mM CaCl_2_, at 37 °C for 20 h, followed by enzyme inactivation at 95 °C for 10 min. Then 20 µL 35 mM pNP-G2 and 5 µL 500 nM *Hv*LD were added to reach the same assay conditions as above. The reaction ran for 2 h at 37 °C, followed by 95 °C for 10 min.

Six alternative glucoside compounds and an alloside compound (Figure 1) were tested as acceptors for TG by *Hv*LD with G1G3G3 as donor. Different acceptor concentrations were used in the assays due to differences in water solubility. The assay concentration of each compound was 14 mM (arbutin, salicin, and raspberry ketone glucoside) or as high as possible (1.6 mM esculin; 1.8 mM 4-methylumbelliferyl β-d-glucoside; 5.2 mM 4-formylphenyl β-d-allopyranoside; and 8 mM 2-formylphenyl β-d-glucopyranoside). The reactions were run as described for G1G3G3 with the pNP compounds.

Some samples were initially analyzed by TLC: 2 × 2 µL or 3 × 2 µL. Samples, including blank samples (as above but without enzyme), were spotted on silica gel 60 TLC plates (Merck, Darmstadt, Germany). A mix of glucose (2 mM) and linear maltooligosaccharides (G2 to G7; 2 mM of each) was included as a standard (1 × 2 µL spot). The separation was carried out in 1-butanol/ethanol/MilliQ water (5:5:3, *v*/*v*/*v*) as the mobile phase, and sugars were visualized with 2% (*w*/*v*) 5-methylresorcinol in ethanol/sulfuric acid/MilliQ water (80:10:10, *v*/*v*/*v*) and heat treatment.

Identification and quantification of TG products was performed by LC-ESI-MS on an Amazon SL iontrap (Bruker Daltonics, Bremen Germany) coupled to an UltiMate 3000 UHPLC from Dionex (Sunnyvale, CA, USA). Samples of 100 µL (75% acetonitrile) were injected on an Acquity Premier Glycan BEH Amide column (130Å, 1.7 µm, 2.1 × 150 mm; Waters, Miford, MA, USA). The chromatography was performed at 60 °C on a two-eluent system with eluent A (acetonitrile) and eluent B (50 mM ammonium acetate pH 5). The elution profile was as follows: 0 min, 25% B; 0–35 min, linear gradient to 46% B; 35–36.5 min, linear gradient to 100% B; 36.5–39.5 min, isocratic 100% B; 39.5–43.1 min, linear gradient to 25% B; 43.1–55 min, isocratic 25% B. In addition, a flow gradient was applied to avoid overpressure: 0–35 min, isocratic 0.4 mL/min; 35–36.5 min, linear gradient to 0.2 mL/min; 36.5–43.1 min, isocratic 0.2 mL/min; 43.1–47.6 min, linear gradient to 0.4 mL/min; 47.6–55 min; isocratic 0.4 mL/min. TG products originating from pNP-sugars were quantified by UV absorption at 302 nm and expressed as pNP-G2 equivalents using external standards. For the alternative acceptors, TG products were detected at the maximum absorbance of the corresponding acceptor. The electrospray was operated in negative mode with UltraScan mode, and a scan range from 100 to 2000 *m/z*, smart parameter setting of 500 *m/z*, capillary voltage at 4.5 kV, end plate off-set 0.5 kV, nebulizer pressure at 3.0 bar, dry gas flow at 12.0 L/min, and dry gas temperature at 280 °C.

### 3.3. Transglycosylation Reactions with Fluoride Donors

To further probe the TG ability of *Hv*LD, reactions including 20–40 mM fluoride compounds (G2F and G3F) as donors and 8–200 mM of different linear (G2, G3, G4, and G6) and cyclic oligosaccharides (α-CD, β-CD, and G2-β-CD) as acceptors were set up. In addition to the linear and cyclic oligosaccharides, the known GH inhibitors, 4-*O*-α-D-glucopyranosylmoranoline (G1M) and acarbose, were also used as acceptors, with G2F as donor. The donor to acceptor ratio was 2:1 (20 mM:10 mM) for G2F/acarbose and 2.6:1 (20 mM: 7.7 mM) for G2F/G1M. All reactions were carried out in 50 mM sodium acetate pH 5.5 at 40 °C using 25–37.5 nM *Hv*LD. To reactions with G2F as donor and α-CD or G2-β-CD as acceptors, 1/12 reaction volume of 60 mM G2F was added after 60, 90, 120, and 180 min, aiming to maintain a constant G2F concentration. Samples were taken at different time points (0–240 min), and the reactions were terminated by the addition of 1/10 volume of 1 M NaOH. The reactions were followed by TLC analysis; 1–4 diluted samples were spotted onto the TLC plate (TLC silica gel 60 F254, Merck) and the plate was run 2 to 3 times in mobile phase (acetonitrile/ethyl acetate/isopropanol/water; 85:20:50:70, by volume). Then, 1 µL 1 mM maltooligosaccharides (G2, G3, G4, and G6) were included as standards, and in some cases, 1 µL 20 mM pullulan standard 1300 (Sigma-Aldrich) was also applied. The carbohydrates were visualized using a stainer containing 2 % (*w/v*) orcinol, 83% (*v/v*) ethanol, and 11 % (*v/v*) sulphuric acid and heat treatment. Selected samples were also analyzed by MALDI-TOF analysis. Samples were diluted in trifluoroacetic acid before being spotted on a pre-spotted anchorchip plate containing pre-spotted matrix (α-cyano-4-hydroxycinnamic acid) (Bruker Daltonics, Bremen, Germany). The analysis was performed at the JIC Proteomics Facility, John Innes Centre, UK.

## Data Availability

The data presented in this study are available on request from the corresponding author.

## Supplementary Materials

The following supporting information can be downloaded at: https://www.mdpi.com/article/10.3390/molecules28104111/s1, Figure S1: Example of transglycosylation (TG) product identification by liquid chromatography electrospray ionization mass spectrometry (LC-ESI-MS); Figure S2: Thin-layer chromatography (TLC) analysis of the *Hv*LD TG reaction with pNP maltoside (pNP-G2) functioning both as donor and acceptor; Figure S3: Dual peaks observed in both UV and extracted ion chromatograms indicating the presence of more than one structural isomer; Figure S4: Extracted UV chromatograms and the corresponding extracted ion chromatogram of TG products using alternative acceptors compared with blank reactions; Figure S5: MALDI-TOF spectrum of TG products from 60 min reaction including G2F as donor and maltose (G2) as acceptor; Figure S6: MALDI-TOF spectrum of TG products from 180 min reaction including α-maltosyl fluoride (G2F) as donor and α-CD as acceptor; Figure S7: TLC analysis of TG reaction using G2F as donor and β-CD as acceptor. Figure S8: MALDI-TOF spectrum of TG products from 120 min reaction including α-maltosyl fluoride (G2F) as donor and 4-O-α-D-glucopyranosyl-moranoline (G1M) as acceptor; Figure S9: MALDI-TOF spectrum of TG products from 120 min reaction including α-maltosyl fluoride (G2F) as donor and acarbose as acceptor.

## Sample Availability

Samples of the most of the compounds used in the study are available from the corresponding author.
