# Supplementary material for: Exploration of the Transglycosylation Activity of Barley Limit Dextrinase for Production of Novel Glycoconjugates
Source: Molecules. 2023 May 16;28(10):4111. doi: 10.3390/molecules28104111 (PMC10223164; doi:10.3390/molecules28104111)
Supplement: Supplementary file 1 [file molecules-28-04111-s001.zip › molecules-2350374-supplementary.pdf]

Supplementary Materials

Exploration of the Transglycosylation Activity of Barley Limit Dextrinase for Production of Novel Glycoconjugates

Malene Bech Vester-Christensen [1], Jesper Holck [2], Martin Rejzek [3], Léa Perrin [4], Morten Tovborg [5], Birte Svensson [1], Robert A. Field [3] and Marie Sofie Møller [4,*]

[1] Enzyme and Protein Chemistry, Department of Biotechnology and Biomedicine, Technical University of Denmark, DK-2800 Kgs. Lyngby (M.B.V-C. present address: Antibody Technology, Novo Nordisk A/S, DK2760 Måløv)

[2] Enzyme Technology, Department of Biotechnology and Biomedicine, Technical University of Denmark, DK-2800 Kgs. Lyngby

[3] Department of Biological Chemistry, John Innes Centre, Norwich Research Park, Norwich NR4 7TJ, UK. (R.A.F. present address: Department of Chemistry and Manchester Institute of Biotechnology, University of Manchester, Manchester M1 7DN, UK)

[4] Applied Molecular Enzyme Chemistry, Department of Biotechnology and Biomedicine, Technical University of Denmark, DK-2800 Kgs. Lyngby

[5] Novozymes A/S, DK-2800 Kgs. Lyngby

* Correspondence: msmo@dtu.dk

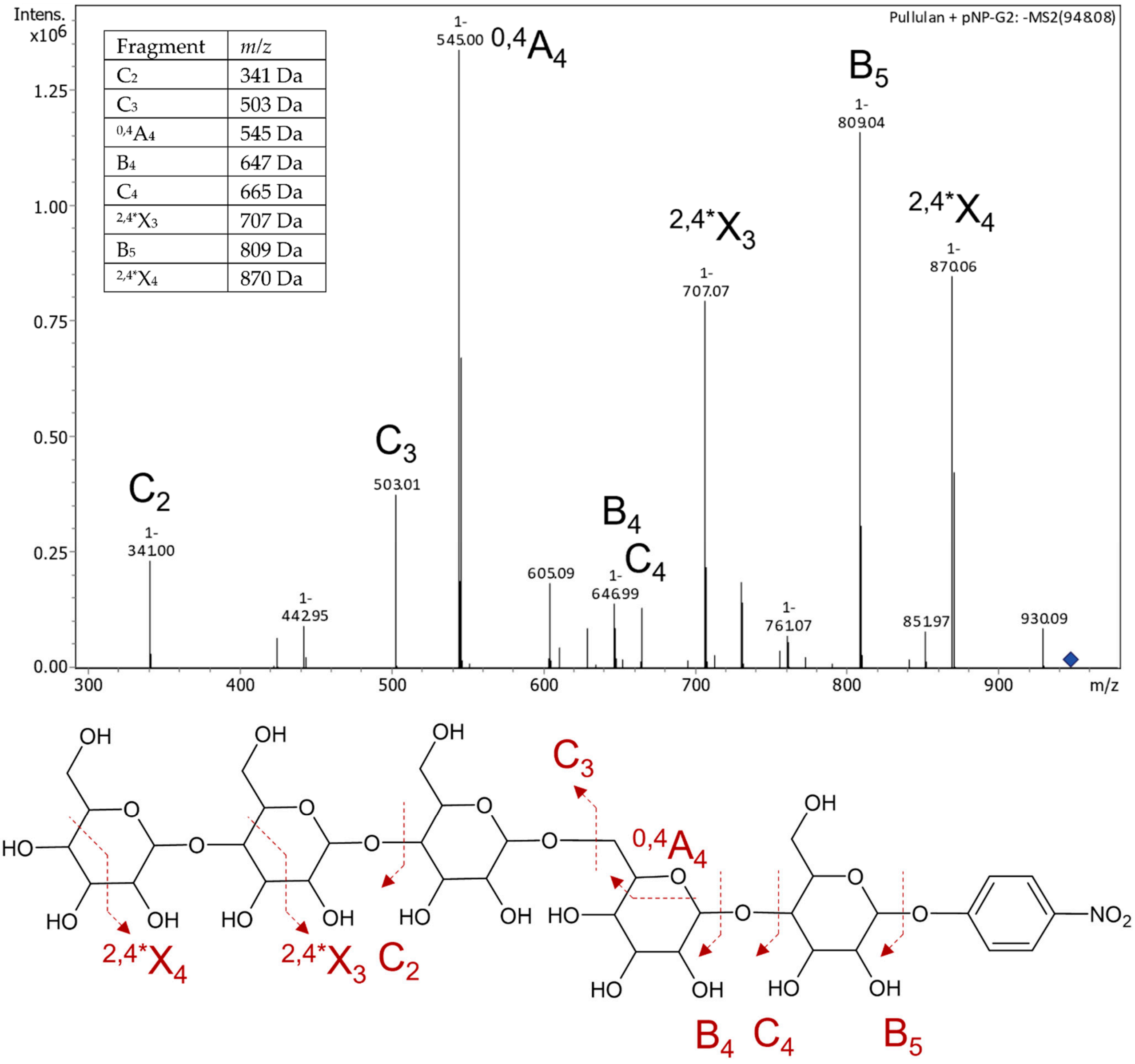

Fragment	*m/z*
C_2	341 Da
C_3	503 Da
$^{0,4}A_4$	545 Da
B_4	647 Da
C_4	665 Da
$^{2,4*}X_3$	707 Da
B_5	809 Da
$^{2,4*}X_4$	870 Da

Figure S1. Example of transglycosylation (TG) product identification by liquid chromatography electrospray ionization mass spectrometry (LC-ESI-MS). Top: MS^2 fragmentation spectrum of *p*-nitrophenyl 6^2-α-maltotriosyl-maltoside (pNP-G5) *m/z* 948.1 Da with identified fragments. Bottom: fragmentation pattern of pNP-G5. Nomenclature was suggested by Domon and Castello.

Domon, B.; Costello, C.E. A systematic nomenclature for carbohydrate fragmentations in FAB-MS/MS spectra of glycoconjugates. *Glycoconj. J.* **1988**, *5*, 397–409, doi:10.1007/BF01049915.

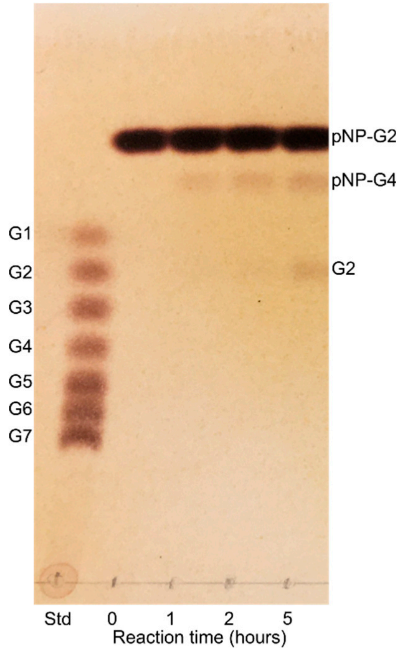

Figure S2. Thin layer chromatography (TLC) analysis of the *Hv*LD TG reaction with pNP maltoside (pNP-G2) functioning both as donor and acceptor. Glucose and maltooligosaccharides (G2 to G7) are included as standards. Released para-nitrophenol (pNP) is not stained by the stainer used.

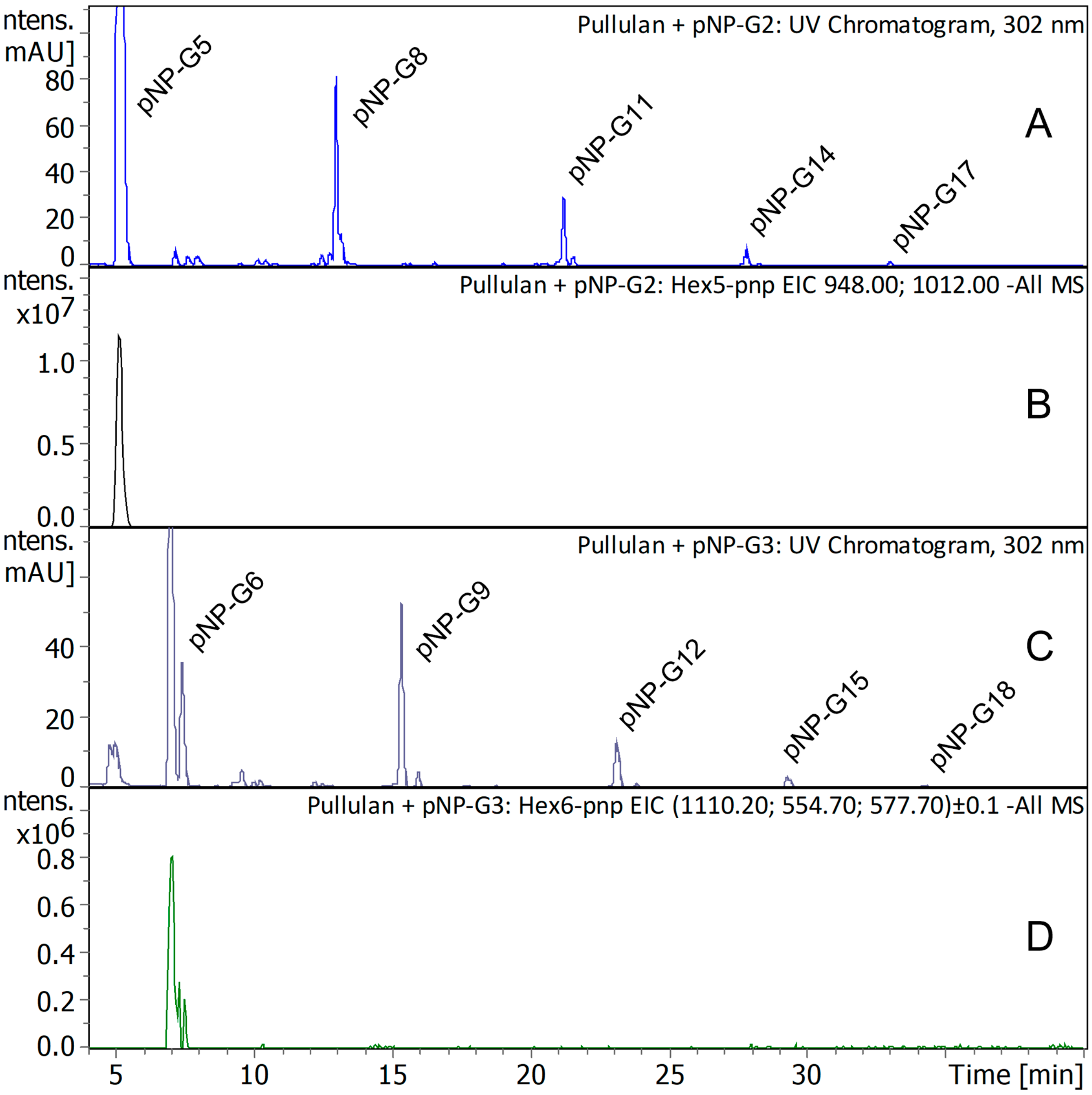

Figure S3. Dual peaks observed in both UV and extracted ion chromatograms indicating the presence of more than one structural isomer. Representative UV chromatograms of reactions with pullulan and pNP-G2 (**A**) or pNP-G3 (**C**). Extracted ion chromatograms of TG products pNP-G5 (**B**) and pNG-G6 (**D**). The TG reaction time was 2 hours.

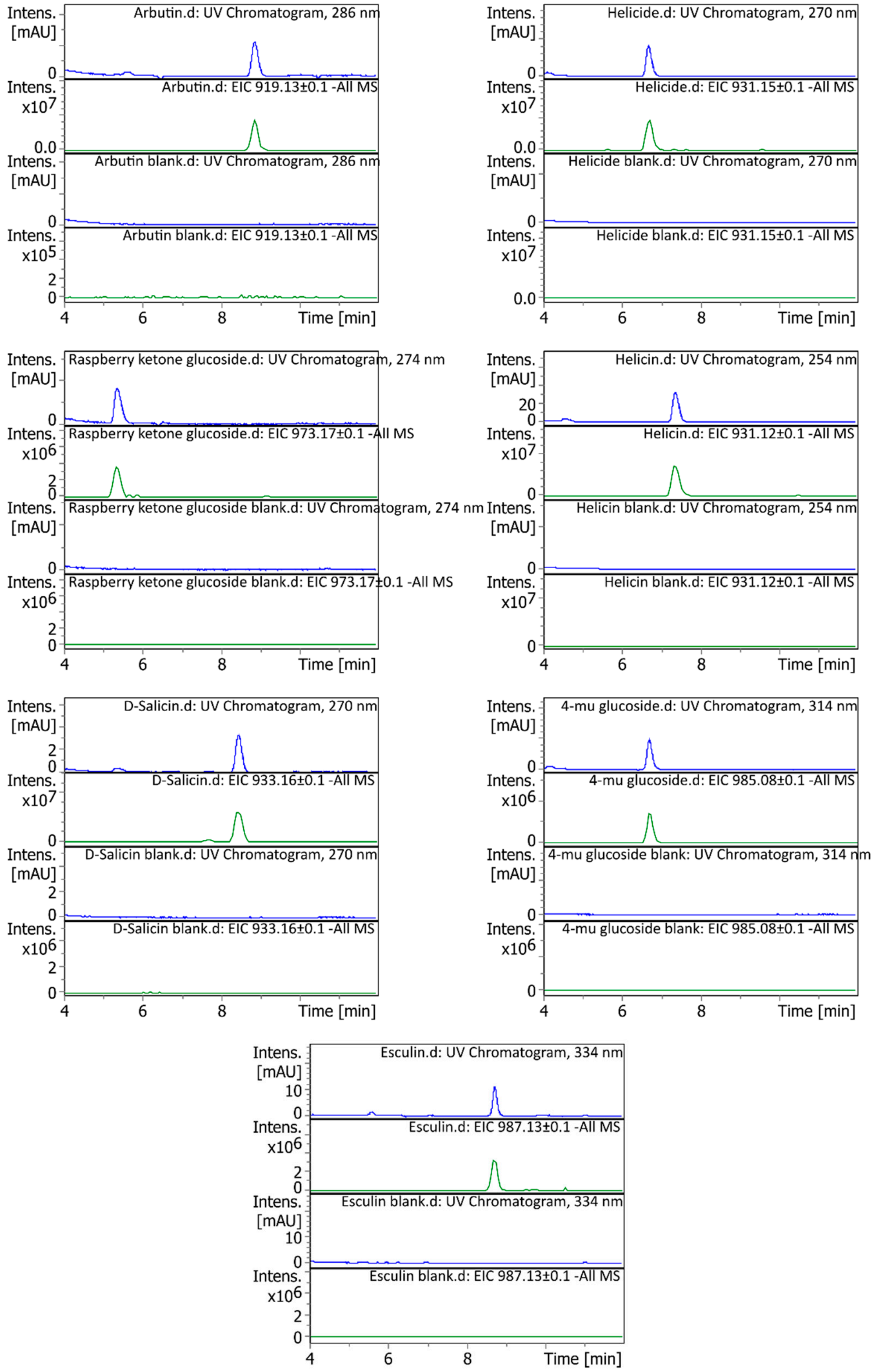

Figure S4. Extracted UV chromatograms and the corresponding extracted ion chromatogram of TG products using alternative acceptors compared with blank reactions. The TG reaction time was 2 hours.

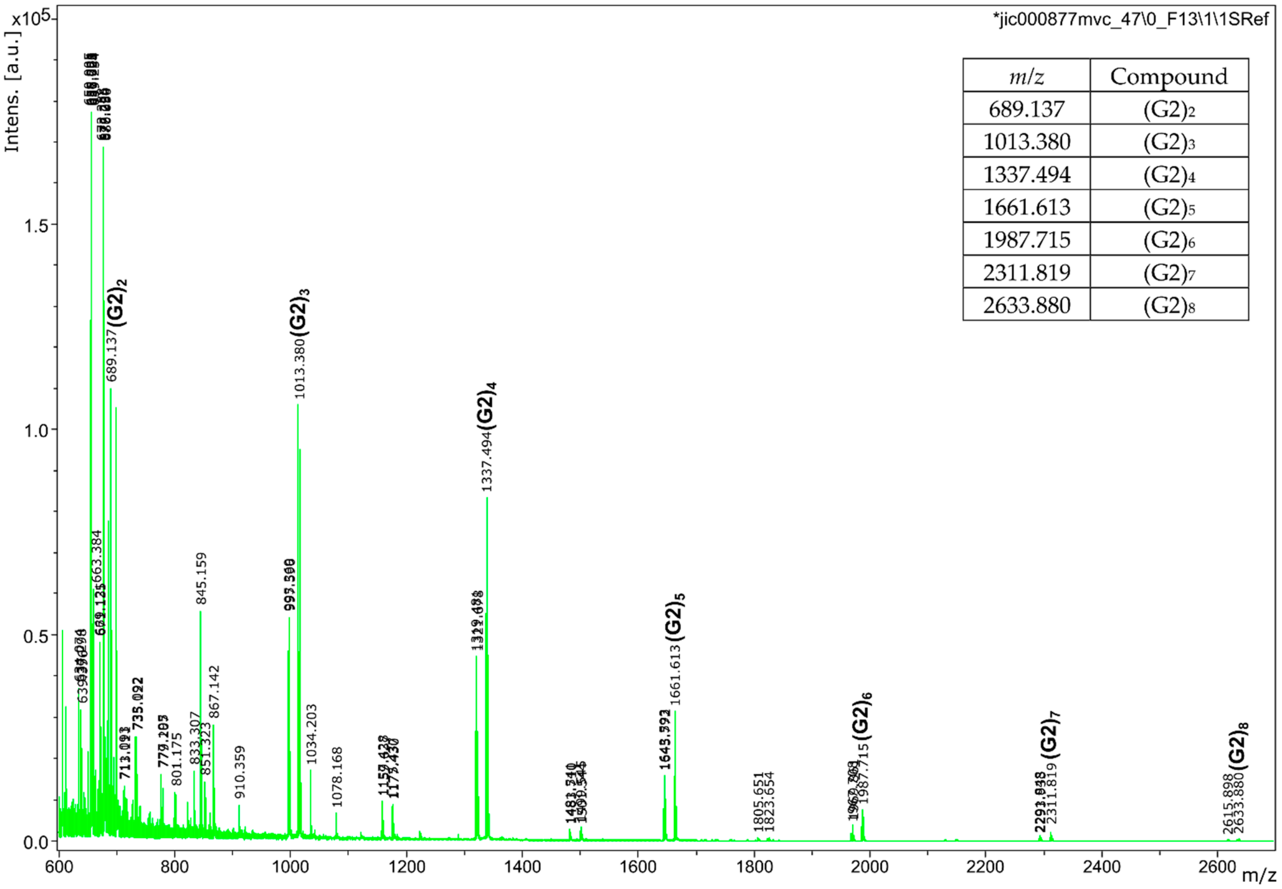

m/z	Compound
689.137	$(G2)_2$
1013.380	$(G2)_3$
1337.494	$(G2)_4$
1661.613	$(G2)_5$
1987.715	$(G2)_6$
2311.819	$(G2)_7$
2633.880	$(G2)_8$

Figure S5. MALDI-TOF spectrum of TG products from 60 min reaction including G2F as donor and maltose (G2) as acceptor.

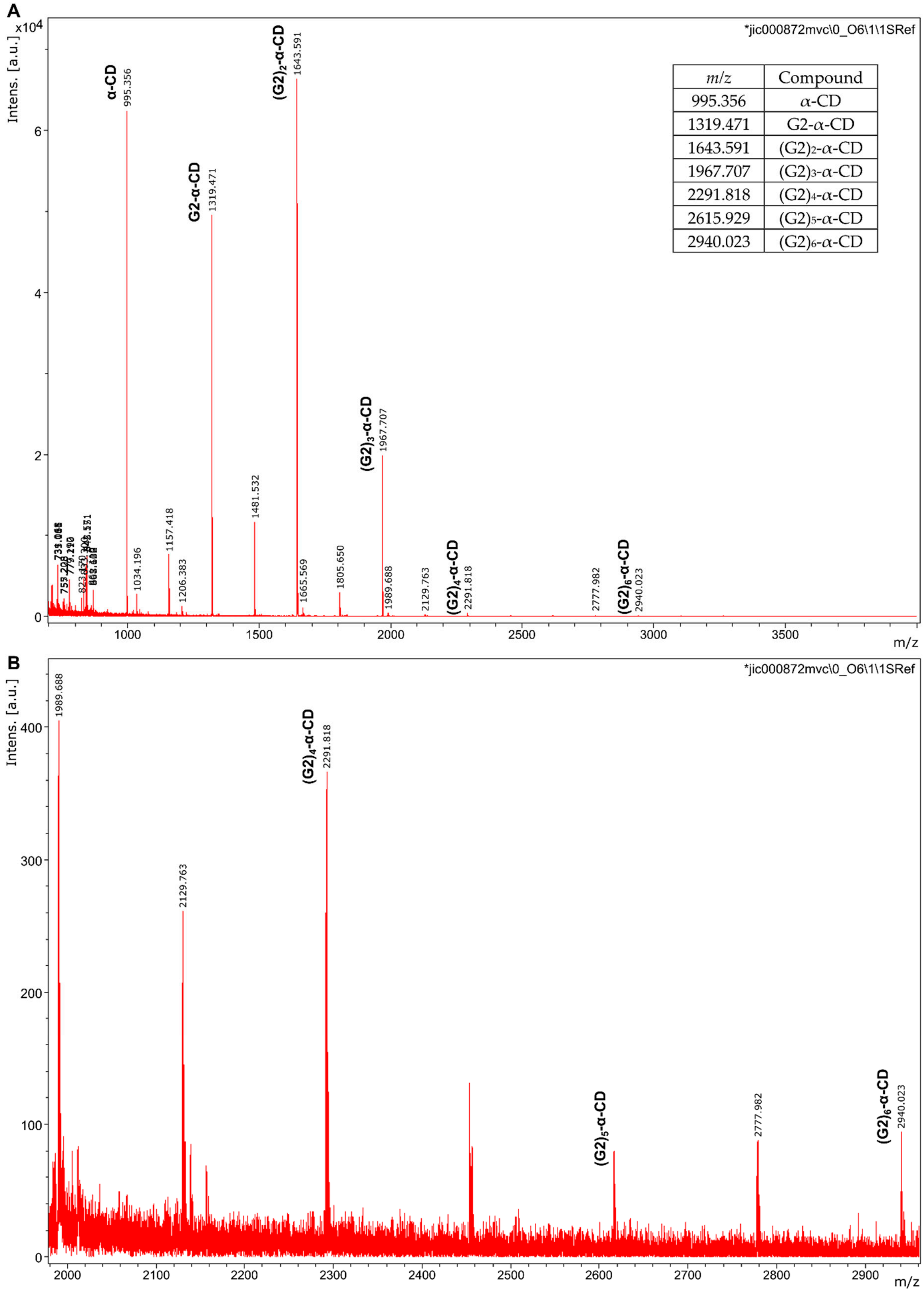

Figure S6. MALDI-TOF spectrum of TG products from 180 min reaction including α-maltosyl fluoride (G2F) as donor and α-CD as acceptor. (**A**) Full range and (**B**) zoomed range of panel **A**.

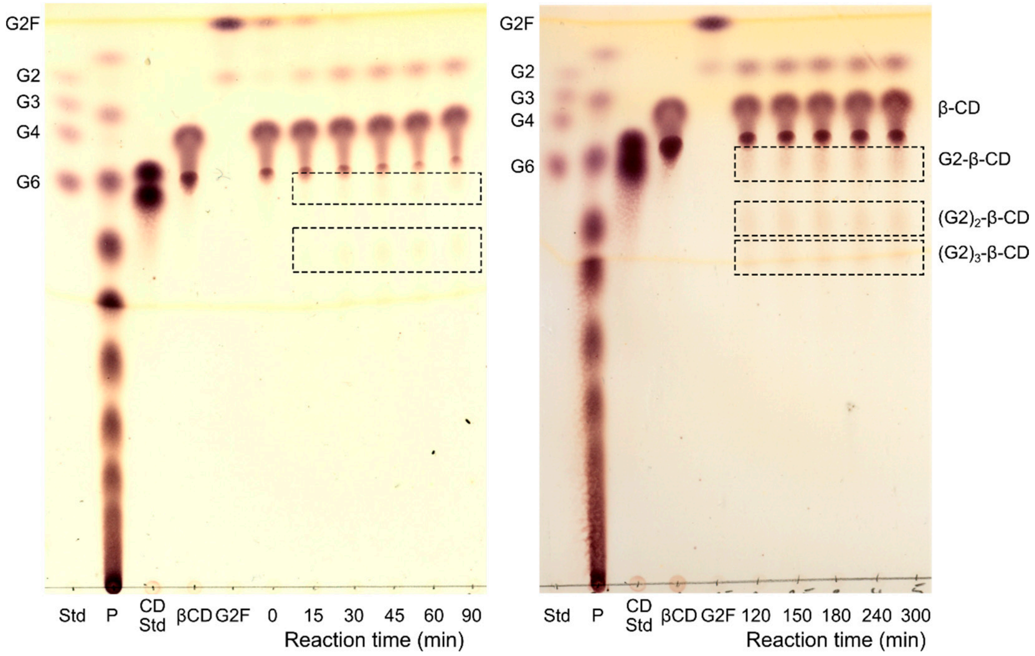

Figure S7. TLC analysis of TG reaction using G2F as donor and β-CD as acceptor. Donor:acceptor ratio of 1:1 (10 mM:10 mM). TG products are framed. Maltooligosaccharides (G2, G3, G4, and G6) are included as standards (Std) together with a pullulan standard (P; see Figure 5 for content) and a mix of G-β-CD and G2-β-CD (CD Std).

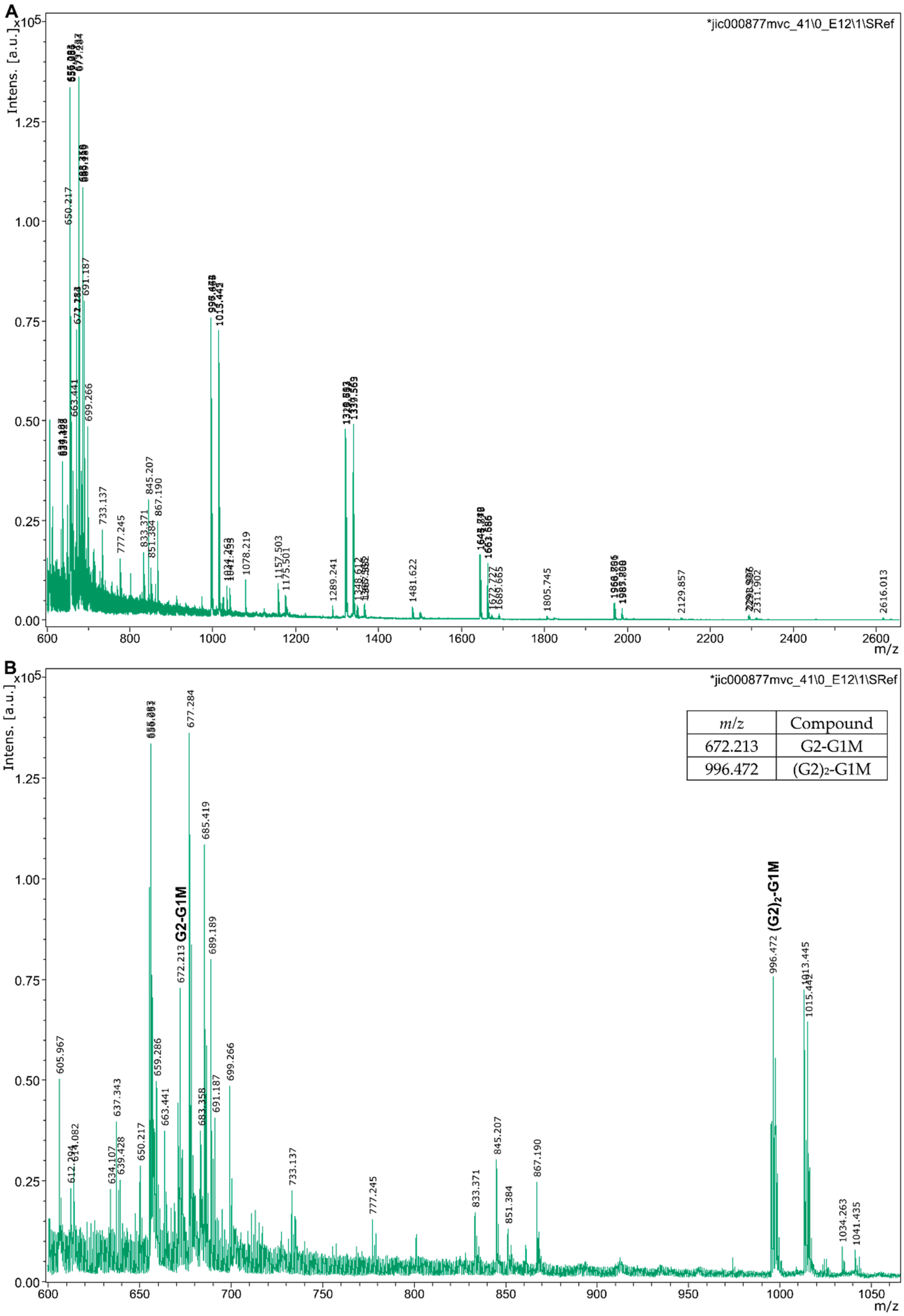

m/z	Compound
672.213	G2-G1M
996.472	$(G2)_2$-G1M

Figure S8. MALDI-TOF spectrum of TG products from 120 min reaction including α-maltosyl fluoride (G2F) as donor and 4-*O*-α-D-glucopyranosyl-moranoline (G1M) as acceptor. (**A**) Full range and (**B**) zoomed range of panel **A** on the lower *m/z* range.

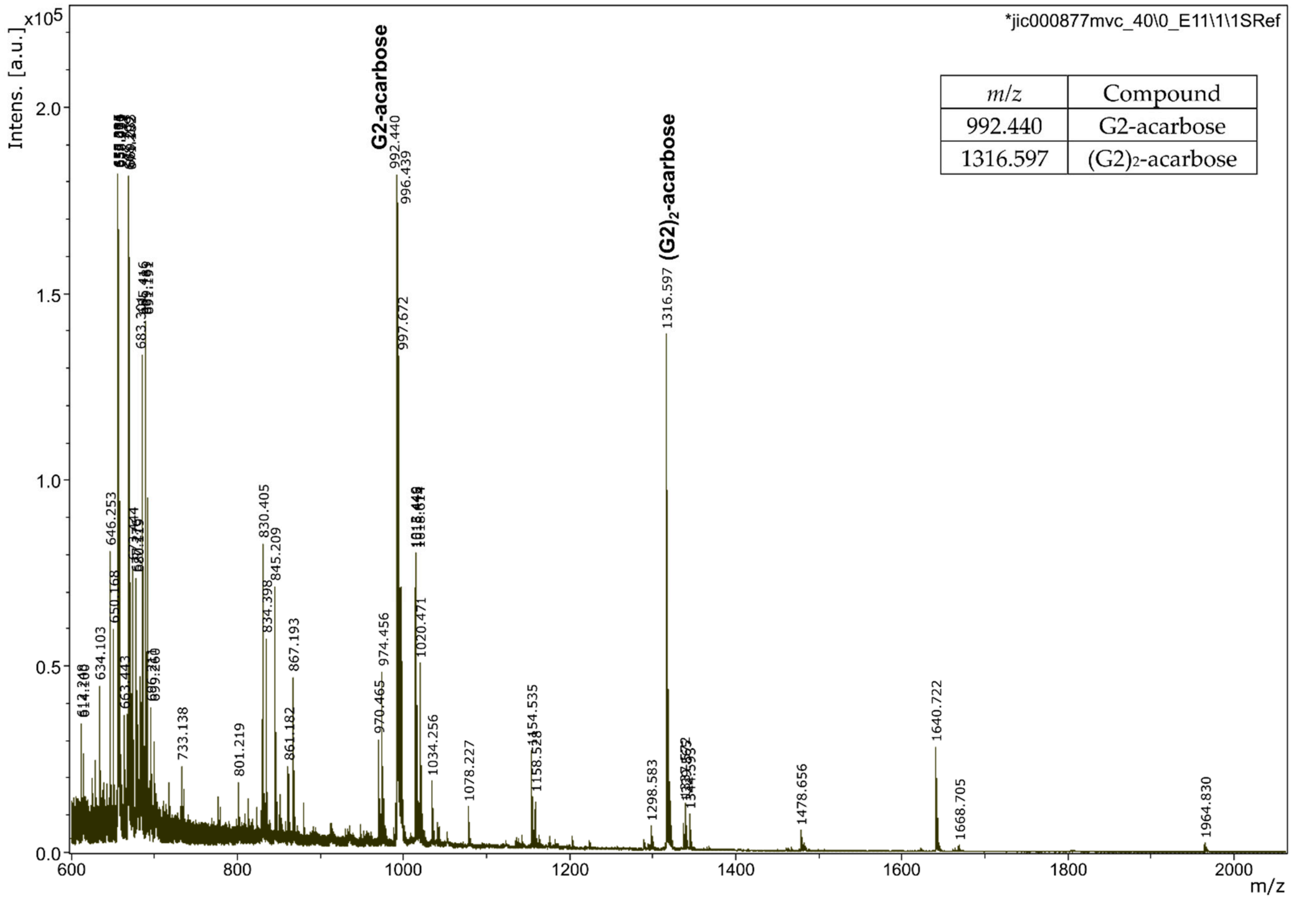

Figure S9. MALDI-TOF spectrum of TG products from 120 min reaction including α-maltosyl fluoride (G2F) as donor and acarbose as acceptor.
